# Later-generation epigenetic aging clocks outperform first-generation models in predicting survival in TCGA breast cancer

**DOI:** 10.1186/s13148-026-02102-3

**Published:** 2026-04-21

**Authors:** Xianglong Tan, Matteo Pellegrini, Su Yon Jung

**Affiliations:** 1 Department of Biological Chemistry, David Geffen School of Medicine, University of California, Los Angeles, Los Angeles, CA 90095 USA; 2 Department of Molecular, Cell and Developmental Biology, Life Sciences Division, University of California, Los Angeles, Los Angeles, CA 90095 USA; 3 Translational Sciences Section, School of Nursing, University of California, Los Angeles, Los Angeles, CA 90095 USA; 4 Department of Epidemiology, Fielding School of Public Health, University of California, Los Angeles, Los Angeles, CA 90095 USA; 5 Jonsson Comprehensive Cancer Center, University of California, Los Angeles, 700 Tiverton Ave., 3-264 Factor Building, Los Angeles, CA 90095 USA

**Keywords:** Epigenetic clocks, Breast cancer, TCGA, Survival analysis, Cox regression analysis

## Abstract

**Background:**

Epigenetic aging bridges the gap between biological and chronological age by exploiting DNA methylation (DNAm) patterns. Over the past decade, successive DNAm-based clocks have been introduced, beginning with the first-generation Horvath and Hannum models and extending to second-generation PhenoAge and the GrimAge family; complementary measures include DNAm-estimated telomere length and mitotic indices such as epiTOC/pcgtAge. We previously conducted a side-by-side evaluation of these metrics in colorectal cancer using publicly available data from The Cancer Genome Atlas (TCGA) COAD and READ cohorts, but an equally systematic assessment in breast cancer has been lacking.

**Result:**

Here, using TCGA-BRCA tumor methylomes linked to clinical data (analytic n = 781), we compared seven metrics (Horvath, Hannum, PhenoAge, GrimAge1, GrimAge2, epiTOC/pcgtAge, DNAmTL) via Kaplan–Meier grouping (median and tertiles) and Cox models adjusted for menopausal status, age at diagnosis, receptor subtype, stage, race, and ethnicity, with overall survival truncated at 4000 days. Our analysis reproduced expected benchmark patterns: Triple Negative Breast Cancer (TNBC) had the worst outcomes, Luminal A the best, and higher stage and older age predicted poorer survival, supporting analytic validity. We found first-generation clocks did not separate survival, whereas PhenoAge and GrimAge2 stratified outcomes; in multivariable analyses, only GrimAge1 provided independent prognostic information. DNAmTL was inversely associated with mortality in univariate models, and epiTOC stratified tertiles but showed wide, nonsignificant Cox estimates.

**Conclusions:**

Second-generation clocks demonstrated stronger prognostic signal than first-generation models in unadjusted analyses. Among them, GrimAge1 retained independent prognostic value beyond established clinicopathologic factors in breast cancer, supporting further external validation with richer covariates to refine clinical utility.

**Supplementary Information:**

The online version contains supplementary material available at 10.1186/s13148-026-02102-3.

## Introduction

Epigenetic aging captures the divergence between an individual’s biological state and their chronological age. DNA methylation (DNAm) based “epigenetic clocks” quantify this process by modeling age from methylation patterns across the genome [1]. First-generation clocks, exemplified by the Horvath and Hannum models, were optimized to predict chronological age from DNAm [2, 3]. They are highly accurate for dating tissues but were originally not designed to capture morbidity or mortality risk [4]. As a result, their prognostic utility in disease settings can be limited because much of their signal aligns with the known effects of chronological age. To address these limitations, later clocks incorporated biology linked to healthspan and mortality. The Levine “PhenoAge” clock integrates DNAm surrogates of clinical chemistry and hematologic markers to approximate phenotypic aging, improving associations with morbidity and survival [5]. The GrimAge family goes further by combining DNAm surrogates for plasma proteins and smoking exposure [6]; the updated GrimAge2 improves the mortality signal and calibration relative to the original GrimAge1 [7]. Related methylation metrics such as DNAm-estimated telomere length (DNAmTL) and indices tied to stem-cell division (e.g., pcgtAge) provide complementary views on cellular aging, replicative history, and systemic stress [8, 9]. Taken together, these metrics bridge age estimation and mortality risk, positioning them for rigorous assessment in oncology cohorts.

Breast cancer is heterogeneous, with outcomes shaped by hormone receptor status, human epidermal growth factor receptor-2 (HER2) amplification, tumor stage, and patient age at diagnosis [10]. The Cancer Genome Atlas (TCGA) provides one of the largest resources linking tumor tissue-based DNAm profiles to curated clinical data in breast cancer, generating a TCGA-BRCA dataset [11]. Although individual clocks have been examined in various cancers, there remains a need for a systematic, side-by-side evaluation of first-, second-, and third-generation DNAm clocks for prognostication in TCGA-BRCA, while accounting for established clinicopathologic determinants. We previously performed such a systematic analysis in colorectal cancer using TCGA-COAD and TCGA-READ, highlighting both the promise and the caveats of DNAm clocks in cancer prognosis [12, 13]. Whether similar patterns hold in breast cancer has not been clarified.

Here, we undertake a comprehensive assessment of multiple DNAm aging metrics in TCGA-BRCA. We compared first-generation clocks (Horvath, Hannum), second-generation PhenoAge (Levine), and the GrimAge family including GrimAge2, alongside DNAmTL and a stem-cell division proxy for their effects on breast cancer survival. Our goal is to determine which DNAm clocks, if any, provide prognostic information beyond standard clinical factors in breast cancer, and to delineate which aging measurement matrices of a clock (e.g., raw value, age-acceleration residual, or age difference) are most informative.

## Materials and methods

### Study population

We retrieved the DNAm dataset of 1076 breast cancer patients from TCGA using the R package *TCGAbiolinks*. Subsequently, the clinical records were retrieved from the TCGA-BRCA “Clinical Supplement (BCR Biotab)” files and merged with curated survival data and immunohistochemistry (IHC) biomarkers (i.e., estrogen receptor/progesterone receptor [ER/PR], and HER2). Patient records were then harmonized by submitter ID and de-duplicated to retain one record per individual. Using an inner join across the DNAm–matched phenotype table, the IHC dataset, and the survival file, we eventually refined the analytic cohort of 781 unique patients for downstream analyses.

We next programmatically derived clinical annotations (Table 1). Menopausal status was defined as post-menopausal (n = 496) or pre/peri-menopausal (n = 195); unknown (n = 90) was retained for descriptive summaries. Receptor subtype was assigned from IHC as Luminal A (n = 309), Luminal B (n = 76), HER2-enriched (n = 17), or Triple-negative breast cancer (TNBC) (n = 83); cases without sufficient IHC were labeled Unknown (n = 296) and excluded from subtype-specific analyses. Tumor stages were harmonized by collapsing AJCC sub-stages to Stage I (n = 126), Stage II (n = 439), Stage III (n = 194), Stage IV (n = 11), with Unknown (n = 11). Demographic variables followed TCGA fields for ethnicity—Hispanic or Latino (n = 38), Not Hispanic or Latino (n = 671), Unknown (n = 72)—and race—White (n = 570), Black or African American (n = 158), Asian (n = 37), American Indian or Alaska Native (n = 1), Unknown (n = 15). “Unknown/not reported” categories were kept in cohort counts and excluded from subgroup comparisons requiring that attribute (Table 1).

**Table 1:** Summary of subject characteristics

Variable	Conditions	TCGA (n = 781)
Menopause Status	Post-menopause	496
	Pre & peri-menopause	195
	Unknown	90
Ethnicity Status	Hispanic or Latino	38
	Not Hispanic or Latino	671
	Unknown	72
Race Status	American Indian or Alaska Native	1
	Asian	37
	Black or African American	158
	White	570
	Unknown	15
Receptor Status	HER2-enriched	17
	Luminal A	309
	Luminal B	76
	TNBC	83
	Unknown	296
Stage Status	Stage I	126
	Stage II	439
	Stage III	194
	Stage IV	11
	Unknown	11

### Preparation of DNAm data: global level of DNAm array

Global level DNAm data was obtained from TCGA. DNA samples derived from breast tissues were analyzed via the Illumina Infinium450K array and normalized via normal-exponential out-of-band (Noob) background correction by *minfi* [14]*.* By using RefFreeEWAS V.2.2 [15], cell-type proportions (cancer and normal epithelial, stromal, and immune cells) of each tumor sample were estimated and adjusted for the heterogeneities in estimating DNAm age. All DNAm clock scores were then computed using the original published coefficients; no model parameters were re-trained, recalibrated or optimized using TCGA-BRCA survival outcomes.

### Survival analysis with demographic factors and cancer characteristics

Overall survival (OS) was evaluated with the *survival* and *survminer* R packages (functions survfit and ggsurvplot). The event indicator was derived from TCGA vital status (Alive = 0; Deceased = 1). To limit undue influence of extreme follow-up, OS time was truncated at 4000 days (OS.time_4000 = pmin(OS.time, 4000)); individuals whose follow-up exceeded 4000 days were censored at 4000 days (event = 0 at that time point). Kaplan–Meier curves were plotted with two-sided log-rank p-values, 95% confidence (95% CI) bands, and risk tables [16].

Groupings for Kaplan–Meier comparisons were done according to the programmatic annotations described in Study Population in Table 1. Specifically, we compared: (i) menopause (pre/peri vs post), (ii) receptor subtypes in prespecified pairwise contrasts (TNBC vs Luminal A; TNBC vs Luminal B; Luminal A vs Luminal B; and HER2-enriched vs each luminal subtype) and collapsed contrasts (Luminal A&B vs TNBC; and Luminal A&B vs HER2-enriched), (iii) tumor stage contrasts (Stage 4 vs others; Stage 1 vs others; Stage 2 vs Stage 3&4; and Stage 3 vs Stage 4), and (iv) demographic groups (by race: Asian vs White; Black or African American vs White; by ethnicity: Hispanic or Latino vs Not Hispanic or Latino). For race-by-ethnicity KM plots, analyses were repeated within each race stratum (White only; Black or African American only; and Asian only). Entries labeled “Unknown” or “not reported” for a given grouping were treated as missing and excluded from that specific comparison.

### Cox regression analysis with demographic factors and cancer characteristics

We modeled OS with Cox proportional hazards using the survival package (coxph). The event‐time object was Surv(OS.time_4000, deceased_4000), where OS was truncated at 4000 days and participants followed longer were censored at day 4000.

For univariate analyses, we fit separate models for each predictor. Age at diagnosis was modeled as a continuous covariate, and hazard ratios (HRs) were interpreted per 1-year increase. Menopausal status was coded as post-menopausal compared with pre/peri-menopausal. For race, White served as the reference and was compared with Black or African American and Asian. For ethnicity, Not Hispanic or Latino served as the reference and was compared with Hispanic or Latino. For receptor subtype, Luminal A served as the reference and was compared with Luminal B, HER2-enriched, and TNBC. For tumor stage, Stage I served as the reference and was compared with Stages II–IV. Categorical variables were explicitly re-leveled to enforce these reference categories. For each model, we reported HRs, 95% CIs, and Wald p values, and displayed the results as log-scale forest plots with a reference line at HR 1.

For the multivariable analysis, we fit a combined model including age at diagnosis, menopausal status, race, and ethnicity, receptor subtype, and tumor stage. Prior to modeling, we excluded records with unknown receptor status, unreported tumor stage, race recorded as not reported or American Indian or Alaska Native, and ethnicity recorded as not reported; unused factor levels were dropped. HRs with 95% CIs and two-sided p values (α = 0.05) were summarized and presented in a forest plot.

### DNAm-based age analysis in association with OS

We evaluated OS in TCGA-BRCA using DNAm–based aging metrics. Categorical covariates were encoded identically to those used in the survival and Cox analyses. OS time and event were defined by OS.time_4000 and deceased_4000.

For each clock metric, in addition to aging scale, we estimated deviation from chronological age, such as DNAmDiff by subtracting chronological age from DNAmAge and age acceleration (AgeAccel) by taking the residual from regressing DNAm age on chronologic age. For Horvath and Hannum clocks, we further derived intrinsic epigenetic age acceleration (IEAA), which represents estimates from DNAm age adjusted for variation in blood cell-type composition and reflects cell-intrinsic aging effects.

Specifically, we analyzed Horvath DNAmAge together with IEAA and DNAmDiff (Horvath DNAmAge minus chronological age); Hannum DNAmAge together with IEAA.Hannum (intrinsic epigenetic age acceleration for the Hannum clock) and DNAmDiff.Hannum (DNAmAgeHannum minus chronological age); and Levine DNAmPhenoAge together with AgeAccelPheno (the residual from regressing DNAmPhenoAge on chronological age) and DNAmDiff.Pheno (DNAmPhenoAge minus chronological age). For GrimAge (both predicted-age GrimAge1 and real-age GrimAge2), we likewise derived an age-acceleration residual and an age-difference measure. DNAm teleomere length (DNAmTL) and the stem-cell division proxy pcgtAge were used on their native scale.

We generated Kaplan–Meier curves under two grouping schemes: a median split (into two groups) and tertiles (into three groups). Curves were compared with log-rank tests; 95% CI bands and risk tables. Univariate Cox proportional hazards models were fit separately for each DNAm aging metric to estimate HRs per unit increase of aging scale, with 95% CIs and Wald p-values. Multivariable Cox models were constructed separately for each DNAm clock family. For the Horvath clock, we fit models with DNAmAge, the age-acceleration residual IEAA, and the age-difference metric DNAmDiff. For the Hannum clock, we used DNAmAgeHannum, the age-acceleration residual IEAA.Hannum, and the age-difference metric DNAmDiff.Hannum. For the Levine clock, we used DNAmPhenoAge, the age-acceleration residual AgeAccelPheno, and the age-difference metric DNAmDiff.Pheno. For GrimAge2, we evaluated the predicted-age and real-age versions, together with their age-adjusted residuals and age-difference metrics. All models were adjusted for menopausal status; age at diagnosis; receptor subtype with Luminal A as the reference; tumor stage comparing Stages II–IV with Stage I while excluding cases coded as Others;race comparing Black or African American and Asian with White while excluding those who did not reported and American Indian or Alaska Native; and ethnicity comparing Hispanic or Latino with Not Hispanic or Latino. Each of the three representations for a given clock was fit in a separate multivariable model.

## Results

### Kaplan–Meier analysis: demographic features associated with patients’ survival

**Fig. 1:**
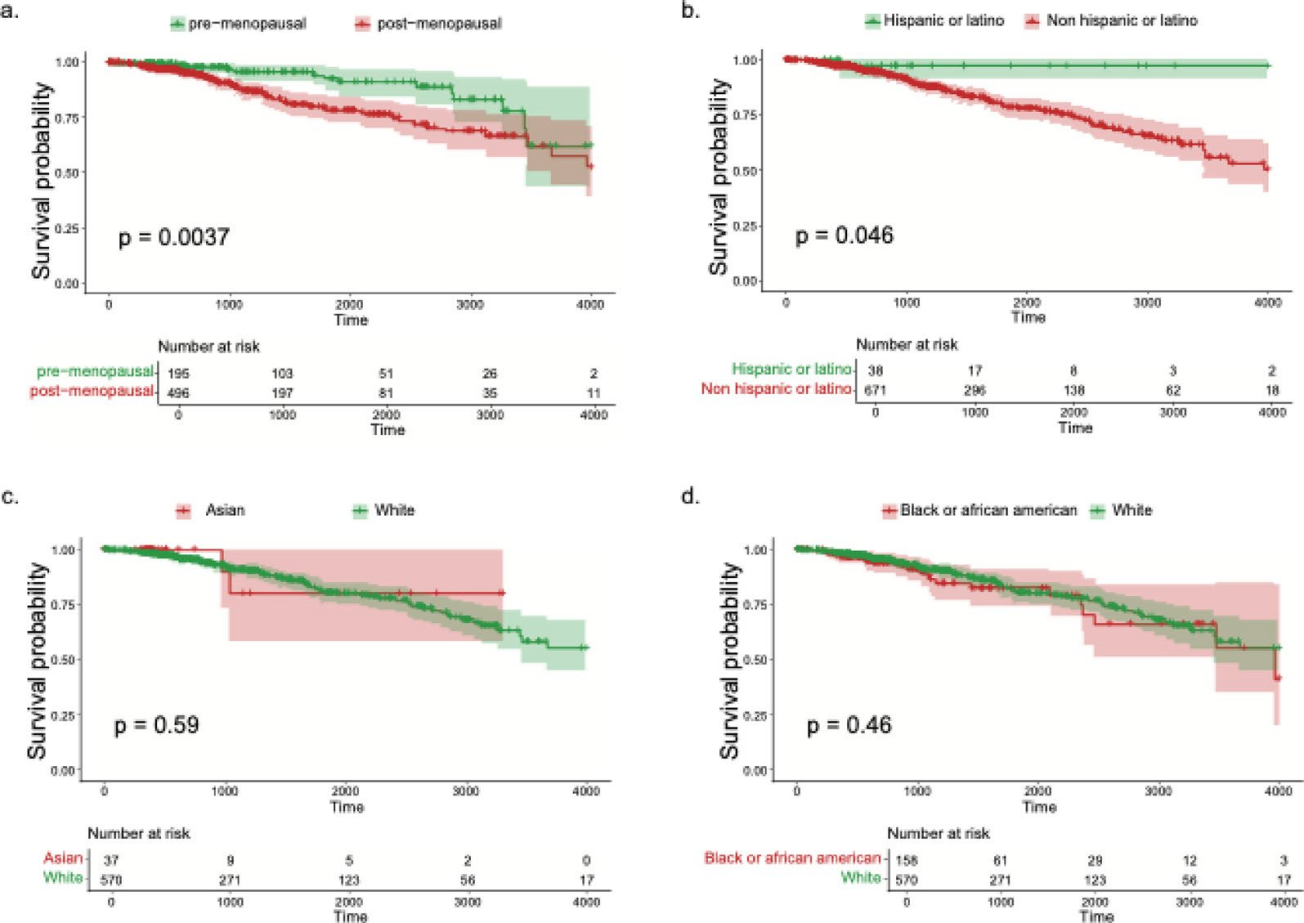
Kaplan–Meier survival curves showing the impact of basic demographic factors on overall survival in TCGA-BRCA patients: **a** Menopausal status (Pre-menopausal vs. Post-menopausal). **b** Ethnicity (Hispanic or Latino vs. Not Hispanic or Latino). **c** Race comparison: Asian vs. White. **d** Race comparison: Black or African American vs. White

To establish baseline survival differences in the TCGA-BRCA cohort, we evaluated demographic and clinical covariates using Kaplan–Meier analyses. Post-menopausal patients exhibited significantly shorter overall survival than pre/peri-menopausal patients (log-rank *p* = 0.0037; Fig. 1a). Ethnicity showed a modest association, with non-Hispanic/Latino patients experiencing worse survival than Hispanic/Latino patients (*p* = 0.046; Fig. 1b). By contrast, race alone (Asian and Black/African American in comparison with White) was not associated with survival (*p* ≥ 0.46; Fig. 1c and 1d). Stratification by combined race and ethnicity categories did not reveal additional differences: White Hispanic/Latino versus White non-Hispanic/Latino showed only a trend (*p* = 0.071), and comparisons within Black/African American and Asian groups were non-significant (*p* ≥ 0.60; Fig. S1). Together, these results indicate that menopausal status is a primary demographic determinant of survival in this cohort, whereas race and ethnicity show limited or no independent associations.

### Kaplan–Meier analysis: receptor status associated with patients’ survival

**Fig. 2:**
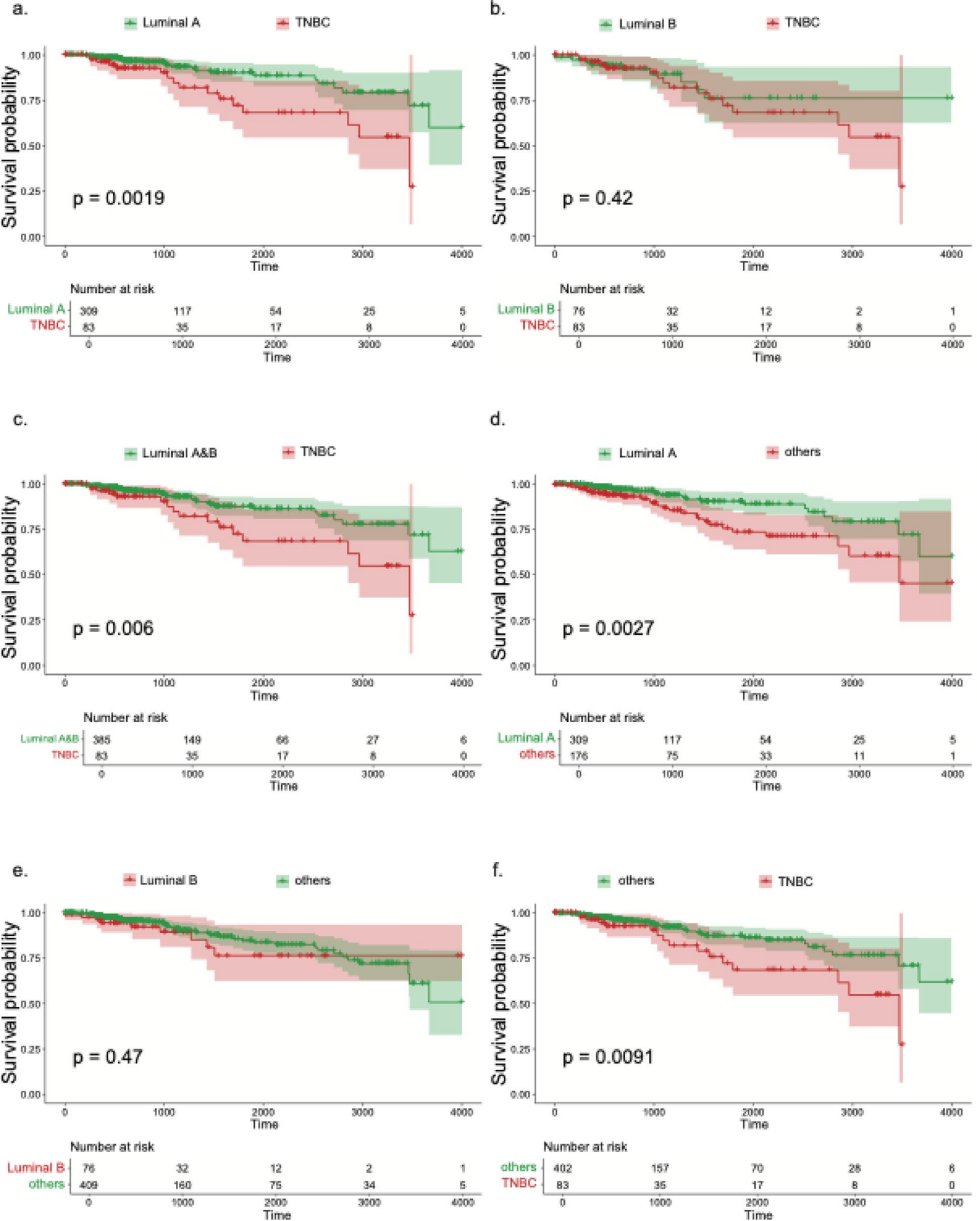
Kaplan–Meier survival curves illustrating the association between receptor subtype status and overall survival in TCGA-BRCA patients: **a** Luminal A vs. triple-negative breast cancer (TNBC). **b** Luminal B vs. TNBC. **c** Luminal A and B combined vs. TNBC. **d** Luminal A vs. all other receptor subtypes. **e** Luminal B vs. all other receptor subtypes. **f** TNBC vs. all other receptor subtypes

We next evaluated baseline receptor-defined subtypes in relation to OS using Kaplan–Meier curves and log-rank tests (Fig. 2, Fig. S2). Luminal A showed markedly better survival than TNBC (*p* = 0.0019; Fig. 2a), and the combined luminal group (A + B) also outperformed TNBC (*p* = 0.006; Fig. 2c), whereas Luminal B alone did not differ from TNBC (*p* = 0.42; Fig. 2b). Luminal A had superior survival compared with all other subtypes combined (*p* = 0.0027; Fig. 2d), while Luminal B versus others was not significant (*p* = 0.47; Fig. 2e). Consistent with these contrasts, TNBC had worse outcomes than the remaining subtypes (*p* = 0.0091; Fig. 2f). Pairwise luminal comparisons were nonsignificant (Luminal A vs B, *p* = 0.12; Fig. S2a). HER2-enriched cases showed no detectable difference versus any comparator (all *p* > 0.05; Fig. S2b–f), noting the small HER2-enriched sample. Collectively, these results position Luminal A as the most favorable and TNBC as the least favorable survival phenotype, with Luminal B intermediate.

### Kaplan–Meier analysis: cancer Clinical stage associated with patients’ survival

**Fig. 3:**
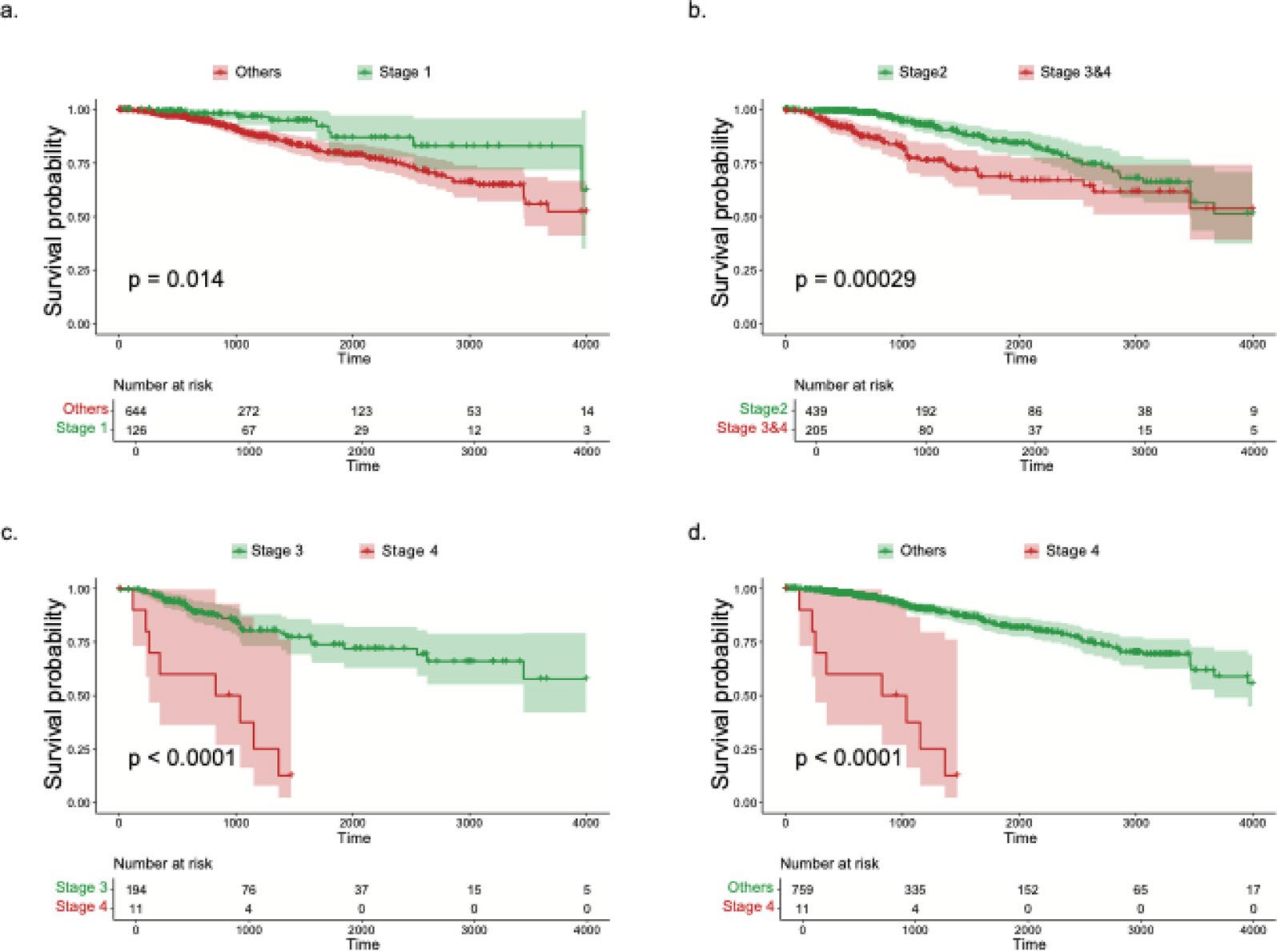
Kaplan–Meier survival curves illustrating the association between earlier tumor stage status versus later stages patients overall survival in TCGA-BRCA patients: **a** Stage 1 versus other stages combined. **b** Stage 2 versus Stage 3 and Stage 4 combined. **c** Stage 3 versus Stage 4. **d** Stage 4 versus other stages combined

Using Kaplan–Meier curves with log-rank tests, we observed a clear stage–survival gradient in TCGA-BRCA. Stage 1 disease showed superior overall survival compared with Stages 2–4 combined (*p* = 0.014; Fig. 3a). Stage 2 also outperformed aggregated advanced disease (Stages 3 and4 combined; *p* = 2.9 × 10⁻^4^; Fig. 3b). Within the advanced disease state, Stage 3 had better survival than Stage 4 (*p* < 0.0001; Fig. 3c). Consistently, Stage 4 had the poorest prognosis relative to Stages 1–3 combined (*p* < 0.0001; Fig. 3d). Together, the curves indicate progressively worse outcomes with increasing stage, with the steepest decline in Stage 4. Of note, times were truncated at 4000 days; cases with missing/indeterminate stages were excluded from stage-specific contrasts.

### Results from inferential analyses for demographic and clinical factors in association with survival

**Fig. 4:**
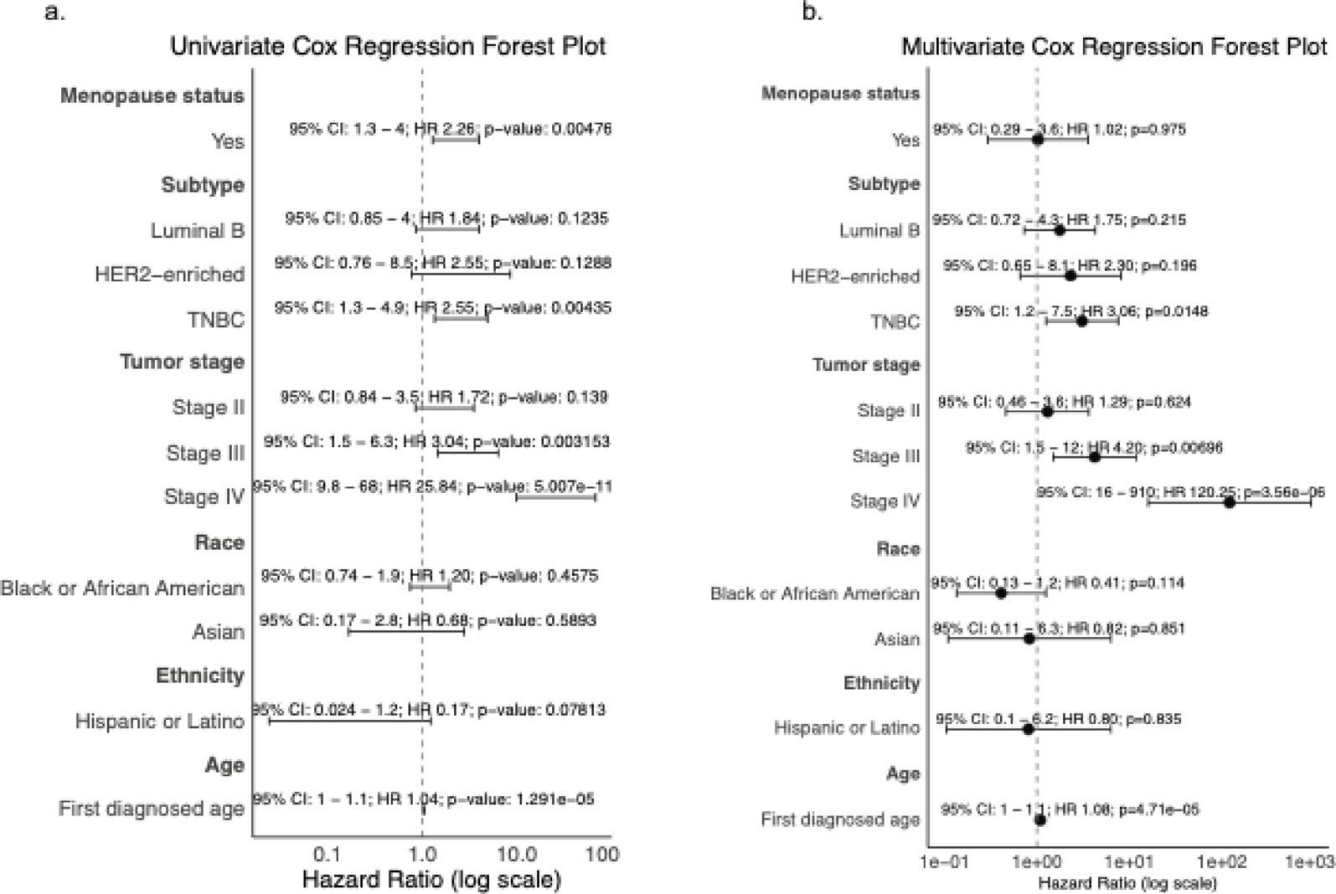
Forest plots of Cox proportional hazards models in TCGA-BRCA: **a** Univariate Cox models evaluating menopausal status, intrinsic subtype, tumor stage, race, ethnicity and age at diagnosis. **b** Multivariate Cox models mutually adjusting for all covariates

To complement the Kaplan–Meier analyses, we fit Cox proportional-hazards models (univariate and multivariable) using pre/peri-menopause, Luminal A, Stage I, White race, and Not Hispanic or Latino as reference categories. Age at diagnosis was modeled as a continuous variable. Forest plots with 95% CIs are shown in Fig. 4a, b. In the univariate analysis, post-menopausal status associated with worse OS (HR 2.26, 95% CI 1.3–4.0, *p* = 0.0048), but the effect disappeared after adjustment (HR 1.02, 95% CI 0.29–3.6, *p* = 0.98), consistent with confounding by tumor features. Relative to Luminal A, TNBC showed a robust increase in hazard in both models (univariate HR 2.55, 95% CI 1.3–4.9, *p* = 0.0043; multivariable HR 3.06, 95% CI 1.2–7.5, *p* = 0.015), whereas Luminal B and HER2-enriched were not significant. Increases in stage strongly tracked with mortality: Stage III and Stage IV remained significant after adjustment (multivariable HR 4.20, 95% CI 1.5–12, *p* = 0.007; HR 120.25, 95% CI 16–910, *p* = 3.6 × 10^−6^, relatively), while Stage II was not. Race and ethnicity showed no association with OS after adjustment (*p* > 0.1). Older age at diagnosis was consistently detrimental, with an ~ 8% higher hazard per year in the adjusted model (HR 1.08, 95% CI 1.0–1.1, *p* = 4.7 × 10⁻^5^). Together, these results reinforce the KM findings and indicate that TNBC status, advancing stage, and older diagnostic age are the strongest correlates of poorer overall survival in this cohort although the very wide CI for Stage IV reflects a small sample size and warrants cautious interpretation.

### Kaplan–Meier analysis: survival analysis of DNAm clocks

**Fig. 5:**
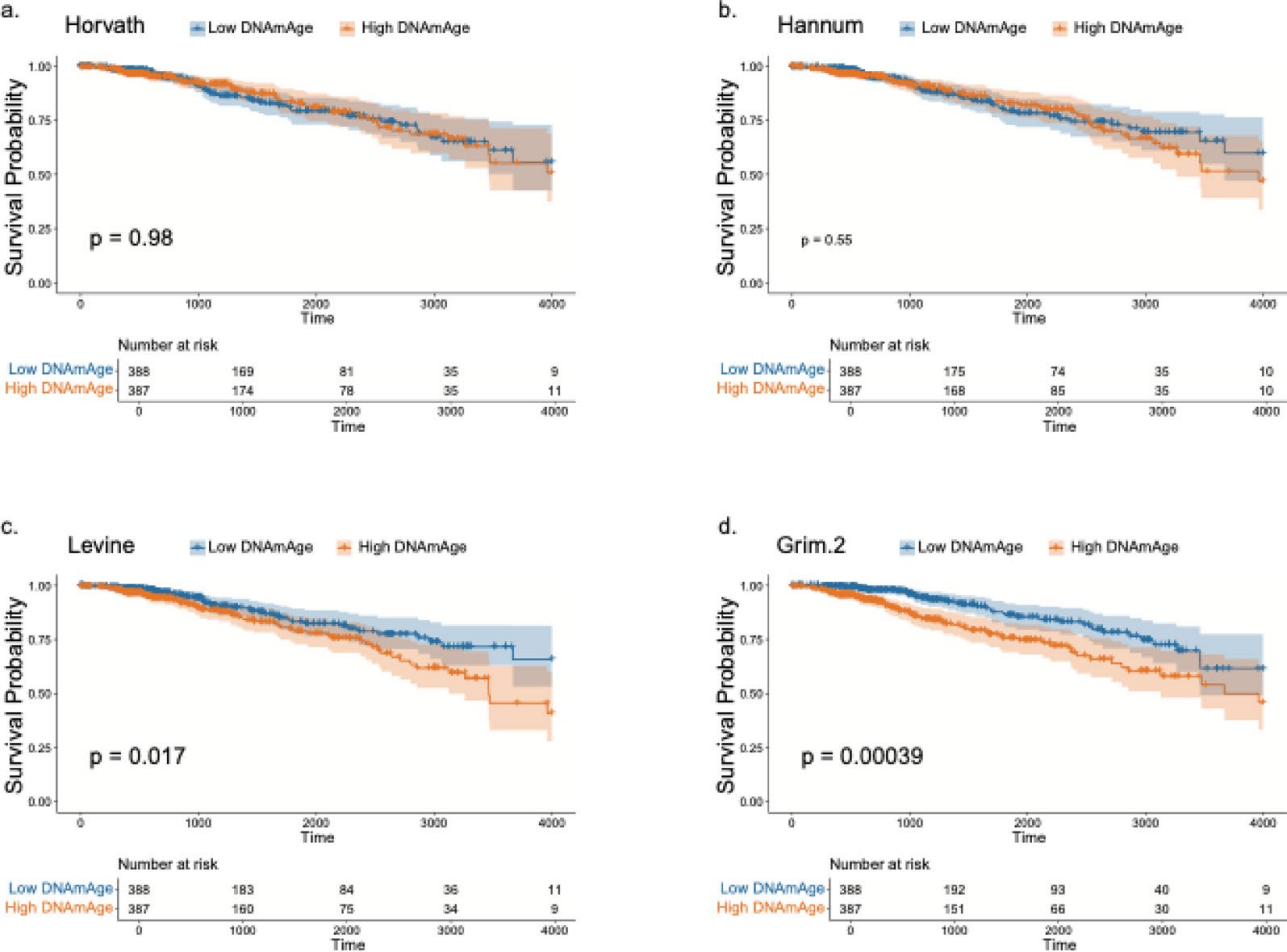
Kaplan–Meier survival curves illustrating the association between DNA methylation–based epigenetic age (DNAmAge) clocks and overall survival in TCGA-BRCA patients: **a** Horvath’s clock. **b** Hannum’s clock. **c** Levine’s clock. **d** GrimAge2 clock

We next used Kaplan–Meier analyses to assess the prognostic value of different DNAm clocks in TCGA-BRCA. Patients were stratified into a binary group by the median value of each clock (Fig. 5; Fig. S3a–c) or into three groups by tertiles (Fig. S3d–j). In addition, models based on DNAmAge residuals and age acceleration measures derived from Horvath, Hannum, Levine, GrimAge1 and GrimAge2 clocks were further examined using median-based dichotomization in Kaplan–Meier analyses (Fig. S3k-t). First-generation clocks (Horvath, Hannum) showed no difference in overall survival between high and low DNAmAge groups (log-rank *p* = 0.98 and *p* = 0.55; Fig. 5a,b). By contrast, higher values from the second generation clocks were associated with worse outcomes: DNAmPhenoAge (Levine) and GrimAge2 each stratified patients into distinct risk groups (Fig. 5c, Levine: *p* = 0.017 and Fig. 4d, GimAge2: *p* = 3.9 × 10⁻^4^). GrimAge1, DNAmTL, and the stem-cell division proxy (pcgtAge) did not yield significant separations (Fig. S3a–c).

Consistent results were observed with tertile stratification: only GrimAge2 produced a graded, statistically significant decline in survival across increasing tertiles (Fig. S3h), whereas other clocks did not show significant differences (Fig. S3d–g,i,j). These findings indicate that later-generation clocks, particularly GrimAge2, provide stronger prognostic discrimination in breast cancer than earlier clocks.

Interestingly, analyses based on age‐acceleration metrics revealed the opposite trend in several models. Specifically, patients in the high acceleration groups defined by the Horvath, Hannum, and GrimAge1 clocks displayed significantly improved overall survival compared with their low-acceleration counterparts (Fig. S3l, n, r). This observation is conceptually consistent with the notion that tumors with more advanced epigenetic aging may be more susceptible to therapeutic eradication, thereby yielding more favorable outcomes.

### Results from inferential analyses for DNAm clocks in association with survival

We further evaluated associations between each DNAm clock and OS using Cox proportional hazards models. For clocks except DNAmTL and stem cell clocks, we independently analyzed three measurement metrics—epigenetic clock value, age‐adjusted residual, and age difference (Table 2). Multivariable models adjusted for menopause status, age at diagnosis, receptor subtype, tumor stage, race, and ethnicity, showing results in forest plots (Fig. S4). In univariate analyses, several clocks as continuous variable showed significant associations with survival. DNAmTL was inversely associated with risk (HR 0.54, 95% CI 0.33–0.89, *p* = 0.0147). Second‐generation clocks were positively associated with risk: Levine (HR 1.01, 95% CI 1.00–1.01, *p* = 0.037), GrimAge1 (HR 1.01, 95% CI 1.00–1.03, *p* = 0.0365), GrimAge2 (HR 1.04, 95% CI 1.02–1.05, *p* = 2.62 × 10^−6^), and the GrimAge2 residual (HR 1.04, 95% CI 1.01–1.06, *p* = 0.0032). Horvath and Hannum clocks, as well as their residual and age‐difference metrics, were not significant. After multivariable adjustment, only GrimAge1 retained independent prognostic value (raw: HR 1.03, 95% CI 1.01–1.06, *p* = 0.0159; residual: HR 1.03, 95% CI 1.00–1.06, *p* = 0.0257; age difference: HR 1.03, 95% CI 1.01–1.06, *p* = 0.0159). Associations for GrimAge2 and Levine attenuated and were no longer significant. In addition, DNAmTL kept showing a negative association with survival, but the trend did not reach significance (HR 0.48, 95% CI 0.20–1.13, *p* = 0.0922). Collectively, while second‐generation clocks capture prognostic signal in unadjusted analyses, only GrimAge1 provides risk information that remains statistically significant, independent of clinicopathologic covariates in TCGA-BRCA.

**Table 2:** Cos regression analyses. Bold values indicate statistical significance (*p* < 0.05)

Epigenetic Clock	Univariate analysis			Multivariate analysis		
	Hazard Ratio	95% CI	*p*-value	Hazard Ratio	95% CI	*p*-value
DNAm.TL	**0.54**	**0.33–0.89**	**0.0147**	0.48	0.20–1.13	0.0922
DNAm.StemCell	6.86	0.97–48.28	0.0531	4.84	0.11–214.78	0.415
Horvath	1	0.99–1.01	0.729	1	0.98–1.02	0.831
Horvath residual	0.99	0.98–1.00	0.245	1	0.98–1.02	0.929
Horvath Age Diff	**0.87**	**0.97–0.99**	**0.0015**	1	0.98–1.02	0.831
Hannum	1.01	1.00–1.02	0.183	1.01	0.99–1.03	0.156
Hannum residual	1.01	0.99–1.02	0.317	1.01	0.99–1.03	0.368
Hannum Age Diff	0.99	0.98–1.00	0.153	1.01	0.99–1.03	0.156
Levine	**1.01**	**1.00–1.01**	**0.0372**	1.01	0.99–1.02	0.296
Levine residual	1	1.00–1.01	0.328	1.01	0.99–1.02	0.296
Levine Age Diff	1	0.99–1.01	0.768	1.01	0.99–1.02	0.296
Grim.1	**1.01**	**1.00–1.03**	**0.0365**	**1.03**	**1.01–1.06**	**0.0159**
Grim.1 residual	1.01	0.99–1.02	0.488	**1.03**	**1.00–1.06**	**0.0257**
Grim.1 Age Diff	0.99	0.98–1.00	0.100	**1.03**	**1.01–1.06**	**0.0159**
Grim.2	**1.04**	**1.02–1.05**	**2.62E-06**	1.03	0.99–1.07	0.179
Grim.2 residual	**1.04**	**1.01–1.06**	**0.00316**	1.03	0.98–1.08	0.293
Grim2. Age Diff	1	0.98–1.02	0.921	1.03	0.99–1.07	0.179

## Discussion

In this study, leveraging the TCGA-BRCA cohort, we systematically evaluated seven epigenetic aging metrics: the Horvath, Hannum, and Levine clocks; GrimAge1 and GrimAge2; a DNAm-based stem-cell division age; and DNAmTL. A key strength of TCGA is the availability of tumor tissue-level DNAm profiles linked to curated clinical annotations, providing a complementary perspective to population cohorts that primarily analyze blood.

Our first examination confirmed expected clinicopathologic survival patterns in TCGA-BRCA. Menopausal status was associated with overall survival in unadjusted KM analyses, with postmenopausal patients showing higher hazard ratios and lower survival probabilities. Race was not associated with survival, whereas ethnicity was—Hispanic/Latino patients experienced significantly better survival than non-Hispanic/Latino patients. Among receptor-defined subtypes, TNBC had the worst overall survival, whereas Luminal A had the most favorable outcomes. Stage-based analyses aligned with clinical expectations, showing progressively poorer outcomes at later stages. Age at diagnosis was inversely associated with survival in Cox models. These observations are consistent with prior reports [17–19] and indicate that our analyses reproduced the expected clinicopathologic survival patterns in TCGA-BRCA, supporting the robustness of our pipeline.

Using DNA methylation data from the TCGA-BRCA cohort, Horvath’s clock is the earliest DNAm-based epigenetic clock and was trained across multiple tissues. Hannum’s clock was developed in blood. Together they are considered first-generation models. In our survival analyses, using continuous values, median splits or tertiles, neither clock measures significantly separated overall survival. Interestingly, in univariate survival analysis, the Horvath age difference was associated with a lower hazard (HR 0.87, *p* = 0.0015), suggesting that in this cohort, higher Horvath’s aging acceleration phenotype corresponded to slightly better outcomes, warranting further validation studies.

Second-generation clocks anchor DNAm CpG features to clinical indicators of morbidity and mortality. The Levine PhenoAge clock integrates DNAm surrogates of composite clinical phenotypic measures and shows improved prognostic performance. GrimAge1 uses a two-stage approach by first deriving DNAm-based surrogates for plasma proteins and smoking exposure, then regressing time-to-death on these surrogates to produce a mortality predictor. GrimAge2 updates the model by adding DNAm estimators for high-sensitivity C-reactive protein and hemoglobin A1c. In TCGA-BRCA, PhenoAge significantly separated survival between high and low groups, and higher PhenoAge was associated with increased mortality in univariate Cox models. Likewise, GrimAge2 stratified survival by median split and showed a strong univariate association with mortality. Together, these findings indicate that second-generation clocks perform better prognostic discrimination than first-generation age-prediction clocks.

Beyond the traditional clocks, Yang et al. proposed a DNAm-based metric approximating a mitotic clock in normal and cancer tissues (epiTOC or pcgtAge in our study) [9]. In the TCGA-BRCA cohort, tertile stratification by epiTOC age revealed a significant survival difference, whereas a median split did not. Although Cox regression analyzing it as a continuous variable did not reach statistical significance with wide confidence intervals, the point estimate suggested a markedly elevated hazard (HR > 4), implying a substantial role in survival prediction in breast cancer patients.

Telomere length is a canonical marker of biological aging and its shortening has been linked to aging and increased cancer risk [20]. In TCGA-BRCA, tertile stratification of DNAmTL showed a significant inverse association with overall survival in which shorter DNAmTL corresponded to poorer outcomes. Our findings are consistent with prior breast cancer studies reporting that short telomeres are enriched in aggressive clinicopathologic subsets and correlate with adverse features (21). In our univariate Cox models, each unit increase in DNAmTL was associated with lower mortality risk, indicating that shorter DNAmTL predicts worse survival.

Although this analysis has the notably strength by leveraging tumor tissue-delivered DNAm profiles from the TCGA dataset, which provides a disease relevant methylome rather than relying on peripheral blood, several limitations should be acknowledged. First, minority racial/ethnic subgroups are under-represented in TCGA-BRCA, reducing statistical power for these subgroup comparisons. Second, clinical and lifestyle epidemiologic annotation is limited and partly dated (e.g., a lack of information about treatment, comorbidity, lifestyle, and socioeconomic variables), constraining multivariable adjustment and leaving room for residual confounding. Finally, this is a single, retrospective dataset with missingness and potential batch effects, warranting external validation. These limitations may attenuate or inflate effect sizes; thus, findings should be cautiously interpreted.

In conclusion, we assessed multiple DNAm-based aging metrics for prognostication in breast cancer in a comprehensive fashion within the TCGA-BRCA data. Second-generation clocks demonstrated stronger prognostic signal than first-generation models in unadjusted analyses. Among them, GrimAge1 retained independent prognostic value after multivariable adjustment. DNAm-based telomere and mitotic clocks showed potential for discriminating survival risk. Prospective validation and integration with richer clinical covariates will be important next steps.

## Supplementary Information

Below is the link to the electronic supplementary material.

Supplementary Material 1.

## Data Availability

The dataset analyzed during the current study are available in TCGA-BRCA repository.

## References

[1] Horvath S, Raj K. DNA methylation-based biomarkers and the epigenetic clock theory of ageing. Nat Rev Genet. 2018;19(6):371–84. 29643443 10.1038/s41576-018-0004-3

[2] Horvath S. DNA methylation age of human tissues and cell types. Genome Biol. 2013;14(10):3156. 10.1186/gb-2013-14-10-r115 PMC4015143 24138928

[3] Hannum G, Guinney J, Zhao L, Zhang L, Hughes G, Sadda S, et al. Genome-wide methylation profiles reveal quantitative views of human aging rates. Mol Cell. 2013;49(2):359–67. 23177740 10.1016/j.molcel.2012.10.016 PMC3780611

[4] Bell CG, Lowe R, Adams PD, Baccarelli AA, Beck S, Bell JT, et al. DNA methylation aging clocks: challenges and recommendations. Genome Biol. 2019;20(1):249. 31767039 10.1186/s13059-019-1824-y PMC6876109

[5] Levine ME, Lu AT, Quach A, Chen BH, Assimes TL, Bandinelli S, et al. An epigenetic biomarker of aging for lifespan and healthspan. Aging (Albany NY). 2018;10(4):573–91. 29676998 10.18632/aging.101414 PMC5940111

[6] Lu AT, Quach A, Wilson JG, Reiner AP, Aviv A, Raj K, et al. DNA methylation GrimAge strongly predicts lifespan and healthspan. Aging (Albany NY). 2019;11(2):303–27. 30669119 10.18632/aging.101684 PMC6366976

[7] Lu AT, Binder AM, Zhang J, Yan Q, Reiner AP, Cox SR, et al. DNA methylation GrimAge version 2. Aging (Albany NY). 2022;14(23):9484–549. 36516495 10.18632/aging.204434 PMC9792204

[8] Lu AT, Seeboth A, Tsai PC, Sun D, Quach A, Reiner AP, et al. DNA methylation-based estimator of telomere length. Aging (Albany NY). 2019;11(16):5895–923. 31422385 10.18632/aging.102173 PMC6738410

[9] Yang Z, Wong A, Kuh D, Paul DS, Rakyan VK, Leslie RD, et al. Correlation of an epigenetic mitotic clock with cancer risk. Genome Biol. 2016;17(1):205. 27716309 10.1186/s13059-016-1064-3 PMC5046977

[10] El Saghir NS, Khalil LE, El Dick J, Atwani RW, Safi N, Charafeddine M, et al. Improved survival of young patients with breast cancer 40 years and younger at diagnosis. JCO Glob Oncol. 2023;9:e2200354. 37229627 10.1200/GO.22.00354 PMC10497296

[11] Comprehensive molecular portraits of human breast tumours. Nature. 2012;490(7418):61–70. 23000897 10.1038/nature11412 PMC3465532

[12] Jung SY, Pellegrini M, Tan X, Yu H. Epigenetic age and accelerated aging phenotypes: a tumor biomarker for predicting colorectal cancer. Aging (Albany NY). 2025;17(7):1624–66. 40632935 10.18632/aging.206276 PMC12339025

[13] Jung SY, Yu H, Tan X, Pellegrini M. Novel DNA methylation-based epigenetic signatures in colorectal cancer from peripheral blood leukocytes. Am J Cancer Res. 2024;14(5):2253–71. 38859857 10.62347/MXWJ1398 PMC11162685

[14] Aryee MJ, Jaffe AE, Corrada-Bravo H, Ladd-Acosta C, Feinberg AP, Hansen KD, et al. Minfi: a flexible and comprehensive Bioconductor package for the analysis of Infinium DNA methylation microarrays. Bioinformatics. 2014;30(10):1363–9. 24478339 10.1093/bioinformatics/btu049 PMC4016708

[15] Houseman EA, Molitor J, Marsit CJ. Reference-free cell mixture adjustments in analysis of DNA methylation data. Bioinformatics. 2014;30(10):1431–9. 24451622 10.1093/bioinformatics/btu029 PMC4016702

[16] D’Arrigo G, Leonardis D, Abd ElHafeez S, Fusaro M, Tripepi G, Roumeliotis S. Methods to analyse time-to-event data: the kaplan-meier survival curve. Oxid Med Cell Longev. 2021;2021:2290120. 34594473 10.1155/2021/2290120 PMC8478547

[17] Toft DJ, Cryns VL. Minireview: Basal-like breast cancer: from molecular profiles to targeted therapies. Mol Endocrinol. 2011;25(2):199–211. 20861225 10.1210/me.2010-0164 PMC3035993

[18] Gao JJ, Swain SM. Luminal a breast cancer and molecular assays: a review. Oncologist. 2018;23(5):556–65. 29472313 10.1634/theoncologist.2017-0535 PMC5947456

[19] Ren C, Gao A, Fu C, Teng X, Wang J, Lu S, et al. The biomarkers related to immune infiltration to predict distant metastasis in breast cancer patients. Front Genet. 2023;14:1105689. 36911401 10.3389/fgene.2023.1105689 PMC9992813

[20] Willeit P, Willeit J, Mayr A, Weger S, Oberhollenzer F, Brandstätter A, et al. Telomere length and risk of incident cancer and cancer mortality. JAMA. 2010;304(1):69–75. 20606151 10.1001/jama.2010.897

[21] Liu M, Zhang Y, Jian Y, Gu L, Zhang D, Zhou H, et al. The regulations of telomerase reverse transcriptase (TERT) in cancer. Cell Death Dis. 2024;15(1):90. 38278800 10.1038/s41419-024-06454-7 PMC10817947

