# Supplementary material for: Later-generation epigenetic aging clocks outperform first-generation models in predicting survival in TCGA breast cancer
Source: Clin Epigenetics. 2026 Apr 21;18:70. doi: 10.1186/s13148-026-02102-3 (PMC13097738; doi:10.1186/s13148-026-02102-3)
Supplement: Supplementary file 1 — Supplementary Material 1. [file 13148_2026_2102_MOESM1_ESM.docx]

Later-generation epigenetic aging clocks outperform first-generation models in predicting survival in TCGA breast cancer

Xianglong Tan^1,5^, Matteo Pellegrini^2^, Su Yon Jung^3,4,5^*

^1^ Department of Biological Chemistry, David Geffen School of Medicine, University of California, Los Angeles, Los Angeles, CA 90095, USA

^2^ Department of Molecular, Cell and Developmental Biology, Life Sciences Division, University of California, Los Angeles, Los Angeles, CA 90095, USA

^3^ Translational Sciences Section, School of Nursing, University of California, Los Angeles, Los Angeles, CA 90095, USA

^4^ Department of Epidemiology, Fielding School of Public Health, University of California, Los Angeles, Los Angeles, CA 90095, USA

^5^ Jonsson Comprehensive Cancer Center, University of California, Los Angeles, Los Angeles, CA 90095, USA

***Correspondence:** Su Yon Jung, Ph.D.

Associate Professor, Translational Sciences Section, School of Nursing

Joint Associate Professor, Department of Epidemiology, Fielding School of Public Health

Jonsson Comprehensive Cancer Center

University of California, Los Angeles

700 Tiverton Ave.

3-264 Factor Building

Los Angeles, CA 90095, USA

Phone: (310) 825-2840

Fax: (310) 267-0413

E-mail: [sjung@sonnet.ucla.edu](mailto:sjung@sonnet.ucla.edu)

**Running Title**: Epigenetic aging clocks in TCGA BRCA survival

**Key words:** Epigenetic clocks, breast cancer, TCGA, survival analysis, Cox regression analysis

**Supplementary Figure Annotation:**

**Figure S1.** Kaplan–Meier survival curves evaluating the association between combined race and ethnicity categories and overall survival in the TCGA-BRCA cohort:

(a) White Hispanic or Latino versus White Not Hispanic or Latino.

(b) Black or African American Hispanic or Latino versus Black or African American Not Hispanic or Latino.

(c) Asian Hispanic or Latino versus Asian Not Hispanic or Latino.

**Figure S2.** Kaplan–Meier survival curves illustrating overall survival differences across receptor-defined subtypes in TCGA-BRCA patients:

(a) Luminal A versus Luminal B.

(b) HER2-enriched versus Luminal A.

(c) HER2-enriched versus Luminal B.

(d) HER2-enriched versus triple-negative breast cancer (TNBC).

(e) HER2-enriched versus Luminal A and Luminal B combined.

(f) HER2-enriched versus all other receptor subtypes combined.

**Figure S3.** Kaplan–Meier survival analyses assessing the prognostic value of DNA methylation–based epigenetic aging metrics in TCGA-BRCA:

(a–c) Binary stratification by median values of GrimAge1, DNAm-based stem cell division age, and DNAm-estimated telomere length (DNAmTL).

(d–j) Tertile-based stratification by Horvath DNAmAge, Hannum DNAmAge, Levine DNAmPhenoAge, GrimAge1, GrimAge2, DNAmTL, and DNAm-based stem cell division age.

(k–t) Binary stratification based on age-adjusted residuals or age-difference measures derived from all seven epigenetic clock models.

**Figure S4.** Forest plots summarizing multivariable Cox proportional hazards models evaluating associations between epigenetic aging metrics and overall survival in TCGA-BRCA, adjusted for menopausal status, age at diagnosis, receptor subtype, tumor stage, race, and ethnicity:

(a–c) Horvath clock models.

(d–f) Hannum clock models.

(g–i) Levine PhenoAge models.

(j–l) GrimAge1 models.

(m–o) GrimAge2 models.

(p) DNAm-estimated telomere length model.

(q) DNAm-based stem cell division age model.

**Supplementary Figures:**
